# Sexual and Emotional Health after Allogeneic Hematopoietic Cell Transplantation: A Comprehensive Review and Guidelines from the Francophone Society of Bone Marrow Transplantation and Cellular Therapy (SFGM-TC)

**DOI:** 10.3390/jcm11051196

**Published:** 2022-02-23

**Authors:** Tamim Alsuliman, Ludovic Jondreville, Caroline Baylet, Marie-Pierre Dann, Natacha De Bentzmann, Marie-Laure Fontoura, Carole Genty, Anne Huynh, Diane Ibled, Ibrahim Yakoub-Agha, Lara Mercier, Catherine Poirot, Sophie Porcheron, Catherine Tourette-Turgis, Jean-Paul Vernant, Dominique Vexiau-Robert, Stéphanie Nguyen

**Affiliations:** 1 Service d’Hématologie et Thérapie Cellulaire, Hôpital Saint-Antoine, AP-HP, Sorbonne Université, 184 Rue de Faubourg Saint-Antoine, 75012 Paris, France; tameemsoliman@gmail.com; 2 Service d’Hématologie Clinique, Hôpital Pitié-Salpêtrière, AP-HP, Sorbonne Université, 47-83 Boulevard de l’Hôpital, CEDEX 13, 75651 Paris, France; ludovic.jondreville@aphp.fr (L.J.); marie-pierre.dann@aphp.fr (M.-P.D.); diane.ibled@aphp.fr (D.I.); jean-paul.vernant@aphp.fr (J.-P.V.); 3 Service des Maladies du Sang, CHU Angers, 4 Rue Larrey, 49000 Angers, France; caroline.baylet@chu-angers.fr; 4 Service d’Hématologie Greffe, IUCT-Oncopole, 1 Avenue Irène Joliot Curie, CEDEX 9, 31059 Toulouse, France; debentzmann.natacha@iuct-oncopole.fr (N.D.B.); huynh.anne@iuct-oncopole.fr (A.H.); mercier.lara@iuct-oncopole.fr (L.M.); 5 Unité de Soins Intensifs Hématologie, CLCC Henri Becquerel, 1 Rue d’Amiens, 76038 Rouen, France; marie-laure.fontoura@chb.unicancer.fr (M.-L.F.); sophie.porcheron@chb.unicancer.fr (S.P.); 6 Service d’Hématologie et de Thérapie Cellulaire, CHRU Dupuytren, 2 Avenue Martin Luther King, 87042 Limoges, France; carole.genty@chu-limoges.fr; 7 CHU de Lille, Univ Lille, INSERM U1286, Infinite, 59000 Lille, France; ibrahim.yakoubagha@chru-lille.fr; 8 Préservation de la Fertilité, Service d’Hématologie, Unité AJA, Hôpital Saint Louis, 1 Avenue Claude Vellefaux, 75010 Paris, France; catherine.poirot@aphp.fr; 9 Médecine Sorbonne Université, Site Pitié Salpêtrière, 91 Bd de l’Hôpital, 75013 Paris, France; 10 Université des Patients, Sorbonne-Université, 91 Bd de l’Hôpital, 75013 Paris, France; catherine.tourette-turgis@sorbonne-universite.fr; 11 Gynécologie Médicale, Hôpital Saint Louis, 1 Avenue Claude Vellefaux, 75010 Paris, France; dominique.vexiau-robert@orange.fr

**Keywords:** sexual life, allogeneic hematopoietic cell transplantation, genital chronic GVHD, SFGM-TC, guidelines

## Abstract

A person’s sexual and emotional life is greatly impacted after allogeneic hematopoietic stem cell transplantation (allo-HSCT). This topic is not addressed very much by patients and caregivers. Physical, endocrine and genital chronic graft versus host disease (cGVHD)-related disorders are multiple and intertwined with psychological disorders. The Francophone Society of Bone Marrow Transplantation and Cellular Therapy (SFGM-TC) has issued recommendations for a better gynecological monitoring of female recipients after allo-HCT. A patient booklet was also offered to patients in the form of questions and answers to facilitate discussions between patients and caregivers and to improve the management of sexual and emotional life after transplant.

## 1. Sexual and Emotional Life Is Often Affected after Allo-HCT and Not Much Addressed

Sexual health, as defined by the World Health Organization, “is an integral part of overall health, well-being and quality of life (…). The sexual rights of all people must be respected, protected and fulfilled” [1]. The consideration of sexual and emotional health is therefore an integral part of the management of allo-HCT patients. It is a positive global approach to sexuality that is not limited to physical aspects or sexual relations themselves, but also includes fantasies, desires, beliefs, practices, eroticism, pleasure, the notion of procreation, the question of self-respect and a relational dimension to others. Various socio-cultural factors can also influence sexuality, such as ethnicity, religion, work and family background [2].

Sexual problems are a frequent complication of allo-HCT and occur in 50% of men and 80% of women [3,4]. A prospective study performed 2–3 weeks before transplant and 1 year after transplant showed a significant decline in overall sexual function in both sexes (*p* < 0.001, *p* = 0.010, respectively), although men scored better than women. The study reported at least one physical sexual problem 1 year after transplant in 47% of men and 60% of women [5]. This was worsened by cGVHD. Recovery from sexual dysfunction tends to happen within two years, but problems may continue for up to 5–10 years post-transplant [6]. In a prospective study following 131 patients over 3 years post-transplant, 50% of patients reported being sexually inactive [3]. These sexual disorders have a strong and negative impact on the quality of life of transplant patients [3]. They are often under-reported by patients themselves [2] and insufficiently addressed by caregivers [4]. For example, 20% of patients reported dissatisfaction with the information they received about complications related to premature ovarian failure (POF), sexual problems and post-transplant infertility [3]. In a study of 273 patients, sexuality was not discussed in 50% of cases, neither pre-transplant nor at 1 or 3 years post-transplant [7]. This subject might be even less discussed with older patients [8]. However, in the context of increasing overall survival, the management of sexual health is important, regardless of age. Indeed, recent treatment modalities, including less toxic approaches such as reduced intensity conditioning, have allowed an older population to benefit from allografting [9,10]. Several articles have shown that this subject is not sufficiently addressed by healthcare teams and by patients themselves [11,12].

## 2. Complexity of Sexual Problems after Allo-HCT

The sexual problems encountered are multiple and interrelated. Thus, it is difficult to dissociate the purely physiological difficulties linked to treatments (conditioning, cGvHD, immunosuppressants, POF) and psychological difficulties (depression, anxiety, phobias, mood disorders, libido, etc.) [13,14]. In an Australian study of 442 adult allograft patients, physical and psychological dysfunctions were closely related [14]. Thus, men reported erectile dysfunction (79%) and decreased libido (62%) and women reported loss of libido (83%), painful intercourse (73%) and less enjoyment of sex (68%). Women also commonly reported vaginal dryness (73%), vaginal narrowing (34%) and vaginal irritation (26%). Age and cGVHD were significantly associated with sexual dysfunction.

The problem of sexuality in allo-HCT patients is complex: sexual difficulties sometimes predate the transplant, or are even independent from the underlying hematological disease. Some caregivers and patients believe that sexual concerns take a back seat to medical treatment [15,16]. Thus, the fear of death or relapse of the hemopathy often alters libido and sexuality and impacts emotional relationships as well as the patient’s ability to project themselves into the future and into life. Previous treatments, fear of infection increased by the prophylactic measures to be applied on discharge and physical isolation during the transplant can lead to emotional isolation and a fear of physical contact when returning home, even with relatives. Lockdowns and social distancing measures during the COVID pandemic may also have accentuated this impression of isolation and great fragility towards the outside world. Additionally, the announcement of sterility or ovarian insufficiency induced by the transplant treatments can have an impact on subsequent sexual life. Indeed, there is an interplay between the notions of fertility/sexuality/virility/femininity. Finally, it is necessary to take into account the patient’s relationship with his or her partner(s), but also the nature and quality of the relationship between the patient and the caregivers (particularly in terms of trust, availability, respect for intimacy, ability to listen, good distance and attention to the various factors of identification).

## 3. Physical Manifestations of Sexual Dysfunctions after Allo-HCT

At the physical level, many factors, notably hormonal or related to GVH, can affect sexual and emotional life after transplantation. Table 1 summarizes the list of possible complications for women and men.

## 4. Genital GVHD

In women, chronic vaginal GVHD is a major problem affecting sexual and emotional quality of life and occurs at a higher frequency than genital cGVHD in men. For example, in Dyer et al.’s study of 421 surviving allograft patients, the rates of cGVHD were 22% in women vs. 5% in men [14], and this rate may be underestimated in both. Gynecological cGVHD is a problem to be addressed and diagnosed early [17]. A Swedish study evaluated 42 women at a median of 80 months after allo-HCT [18]. Genital cGVHD was diagnosed at a high frequency in 22 out of 42 patients (52%). Its presence was associated with systemic corticoid steroid treatment of extragenital cGVHD, older age and HSCT from a sibling donor. Only five patients had isolated genital cGVHD. Dryness, pain, smarting pain and dyspareunia were observed more frequently in women with genital cGVHD. Twelve patients had advanced genital cGVHD (clinical score 3). Genital cGVHD was common, often associated with extragenital cGVHD and in many cases incorrectly diagnosed. The same group studied 41 women who were examined before and at various times up to 36 months post-allo-HCT [19]. Genital cGVHD was diagnosed in 27 women mainly in the first year after allo-HCT (56% at 12 months; 66% at 36 months) with extragenital cGVHD in most cases (21/27). Early intervention could halt its progress to severe fibrosis, but despite correct diagnosis and treatment, symptoms and signs may also become chronic, as 12 women still had genital cGVHD at 36 months. These findings were in agreement with the observations reported previously by a French study of 32 women with genital cGVHD, which showed that early diagnosis is important to avoid severe complications [20]. At presentation, most patients complained about vaginal dryness and dyspareunia with impairment in sexual activity. Half of the patients had grade I genital lesions and half had grade II or III lesions. Patients seen later in gynecological consultation had more severe lesions than patients seen early after transplantation. Importantly, at the time of diagnosis, most patients had other cutaneous or mucous localizations of cGVHD pointing to the necessity of proposing an early specialized gynecological consultation, especially in case of other cutaneous or mucous localizations of cGVHD, in particular, the mouth, which is very often associated with a gynecological location of GVHD. Local treatment associating steroids and estrogen seemed to prevent further evolution of grade I genital lesions and to avoid surgical treatment, and most patients could resume sexual activity. Treatment was more efficient in patients with mild lesions than in others.

Chronic genital GVH can also affect men [21]. A study evaluated 155 male patients 1 year or more post-transplant, with a median time between allo-HCT and genital examination of 5.9 years (range, 1 to 30.3 years). Thirty-one of 155 patients (20%) had genital skin changes. Interestingly, patients with inflammatory genital skin changes had a significantly higher coincidence of oral (*p* < 0.0001), ocular (*p* < 0.002), and/or cutaneous cGVHD (*p* < 0.026) when compared with patients without genital lesions. Erectile dysfunction was significantly more frequent in patients with genital cGVHD. cGVHD was associated with more frequent erectile dysfunction and sexual discontent. The authors conclude that genital skin changes in male recipients after allo-HSCT are frequent and seem to be an under-reported, relevant late effect. Inflammatory genital skin changes can be considered as a form of genital cGVHD frequently associated with manifestations of extragenital mucocutaneous cGVHD, as observed in female patients.

## 5. Endocrine Dysfunction Post-Allo-HCT

Disease and treatments in the context of allo-HCT also have a great impact on ovarian function and family planning initiatives. In a recent study, 63 adult female patients who underwent allo-HCT before the age of 35 years were followed for more than 2 years [22]. Symptoms of hypoestrogenism were reported by 86% of the patients and changes in sexual life were reported by 76%, mostly because of low sex drive, the negative impact of infertility problems, physical sequelae and loss of self-confidence. POF occurred in 74% of the patients and was significantly associated with conditioning regimen (MAC versus RIC; *p* = 0.001) and baseline disease (bone marrow failure versus acute leukemia versus others; *p* < 0.001). However, half of the patients developed a POF despite the use of a RIC regimen. For 27 patients (47%), disease and treatments modified their desire for pregnancy, mainly due to the fear of relapse and of disease transmission to offspring.

Endocrine dysfunction, especially hypogonadism, in male patients who were allotransplanted in childhood is well described in the literature. However, the impact on sexual life in adulthood is less well known. A study of 97 male survivors of childhood allo-HSCT in Denmark and Finland found, compared to a healthy control group (*n* = 56), fewer sexual fantasies, poorer orgasms, lower sexual activity with a partner and reduced satisfaction with their sex life, even in the presence of normal erectile functions and a similar frequency of autoerotic acts. Risk factors for poorer self-reported sexual functions were partner status (not cohabitating with a partner), depressive symptoms, central nervous system and testicular irradiation. Male patients who received an allo-HCT in adulthood also have impaired testicular, hormonal and sexual function. A recent study conducted in 105 adult male allograft patients assessed the hormonal profile (testosterone, follicle-stimulating hormone, luteinizing hormone, and inhibin B) and the International Index of Erectile Function (IIEF-15) questionnaire, and revealed a higher prevalence of hypogonadism (21%), impaired spermatogenesis (87%) and erectile dysfunction (72%) compared to the general population [23]. cGVHD, especially moderate/severe grade, was associated with an increased risk of developing erectile dysfunction. Moreover, a high proportion of patients had alterations in all domains of sexual function, even after complete clinical remission of hematologic disease. Hormonal deficits in men after allo-HSCT are poorly explored and the value of testosterone replacement therapy is not yet well established. However, this point does deserve to be investigated [24].

## 6. Physical and Psychological Manifestations

In line with these multiple causes, the manifestations of sexual and emotional disorders are also multiple, both psychological and/or physical [16,25,26].

On a psychological level, manifestations may include stress, anxiety, depression, lowered self-esteem, changes in self-image, fears, hyper-vigilance, inability to let go and changes in relationships as a result of the disease. On a physical level, factors that can affect sexual and emotional life can be weight loss or gain (possibly aggravated by immunosuppressants), hair loss, ciclosporin or corticosteroids-associated hyperpilosity, vaginal dryness in women, sterility, dryness of the mucous membranes in men, hormonal dysfunctions, a feeling of great persistent fatigue and lack of energy.

## 7. SFGM-TC Guidelines

### 7.1. Methodology

These recommendations were established by a group of multidisciplinary experts (psychologists, coordinating and/or monitoring nurses, transplant doctors, gynecologists, a doctor specialized in reproduction, a doctor specialized in therapeutic education, a sexologist) taking into account the recommendations established by specialized societies, transplant centers, data from the literature and recent presentations from various conferences [27,28]. The SFGM-TC guidelines are composed of two parts: the first part proposes a gynecological follow-up (Annex S1 in Supplementary Materials), and the second part consists of a questions and answers booklet that can be given to patients (Annex S2 in Supplementary Materials).

### 7.2. Follow-Up of Allo-HCT Patients

A first consultation is recommended before allo-HCT. This consultation aims mainly to assess, document and verify the following clinical and paraclinical aspects. General criteria: age, height, weight, body mass index (BMI), initial disease, thrombo-embolic history, type of conditioning regimen and date of initiation, ferritinemia, complete blood count, coagulation assessment, bone densitometry (dual-energy X-ray absorptiometry), thyroid assessment: TSH, T3L, T4L, anti-thyroglobulin (preferable), fertility consultation and vaccination status. For women: age of first menstruation, regularity of periods, initial or secondary amenorrhea, hormone replacement therapy, childbirth, pelvic infections, gynecological examination, pelvic ultrasound, HPV test, hormonal assessment (FSH, LH, estradiol, prolactin, anti-Müllerian hormone) and antral follicle count (AFC). If the consultation takes place before the allo-HSCT, it is crucial to ask for contraception status. It is also worth noting that the previous items are only relevant in patients who are not on oral contraception and have not started chemotherapy. For men: testosterone, spermogram, search for genital infections (history or active) and history of paternity. Consultations during or after the allogeneic transplant should be organized as needed—for example, after discharge from hospital, and then once a year (see Annex S1 in Supplementary Materials).

### 7.3. Questions and Answers of the Patient’s Booklet

Twelve questions that could be asked by the patient and/or caregivers were chosen by the participants in order to cover as many topics as possible.

These questions are listed with the corresponding answers in italics as they are printed in the patient booklet and given to the patient (see Annex S2 in Supplementary Materials). Scientific references or author comments intended for caregivers are added after the patient response. This article, accompanied by the patient booklet in the appendix, aims to facilitate the caregiver’s or the patient’s understanding of the question, as well as to propose a more systematic and adapted organization of consultations and care before and after transplant.

1. Can I resume intercourse? When can I start having sex again?

*Sexual intercourse can be resumed as soon as you return home. It is important to emphasize that sex is not limited to penetration. It is also about sensual caresses, tenderness, touching, communication, not forgetting masturbation and kissing. Patients often mention a period of gradual resumption of sexual life after the disease.*

If patients respect the hygiene instructions and the standard antibacterial, antiviral and antifungal prophylaxis that have been provided to them at the end of the transplant, and if there are no active infections in the partner, there is no justification for a restriction on physical contact, nor is there any specific time limit to be observed.

2. I am afraid to resume sexual relations. Is this normal?

*After a long period of hospitalization and isolation, associated with very strict hygiene measures, returning home can be a source of fear, questioning and anxiety, which can impact your intimate life. An adaptation phase is often necessary after a cessation of sexual activity while going through such an intense process as a transplant. Communication with your partner is essential. Ask your medical team questions if your fear persists and becomes a major concern for you. No one can imagine what is going on in your head and in your partner’s head. Just talk about it. There may be new forms of erotic and sexual relationships that may be temporary; things are never set in stone.*

The resumption of sexual intercourse is not medically contraindicated; however, it can be adapted to the physical (fatigue, mucositis, etc.), biological (thrombopenia), immunological and psychological state of the patient [29,30,31]. Patients often refer to a period of gradual resumption of sexual life after the illness. However, it may be desirable to prepare the mucous membranes using local estrogenic treatments in patients to facilitate intercourse with penetration (gynecological consultation) [27]. The patient should be notified that the gynecological consultation is also open to the partner, who can thus ask questions about the resumption of the relations and hear the explanations given, which can reassure them both.

3. Are there any particular infectious risks associated with resuming sexual activity? Do I need to use a condom?

*The risks of sexually transmitted infections (STIs) are the same as in the general population. Thus, it is recommended to use a condom in case of multiple partners or unknown HIV status, as well as in case of a history of genital warts in any partner. It is also recommended that condoms be used for oral or anal sex for as long as immunosuppression lasts. In the event of genital or oral lesions, it is necessary to talk to the attending or referring physician, who will be able to guide you.*

Beyond the transmission of STIs, other types of transmission (CMV, parasite, etc.) have not been sufficiently studied. There is no consensus on how long to wear a condom with a stable partner. The recommended period is usually 3 to 12 months but without a clear scientific rationale [31]. Precautions should apply as in the general population, i.e., use of condoms in case of multiple partners or unknown HIV status, or a history of genital wart without time limit in relation to the transplant. Immunosuppression promotes the reappearance of warts, as well as the risk of cervical (need to perform screening smears), penile or even anal lesions in both sexes. Regarding oral or anal intercourse, the condom is recommended because many patients can be infected by healthy carrier companions. There is a significant risk of tonsillar lesions, which are difficult to diagnose. This recommendation remains valid at least as long as the immunosuppression lasts. HPV vaccination should also be considered after allo-HSCT [32,33].

4. Is there a risk of contagion of the cancer to my partner by sexual means?

*No, there is no risk of contagion of cancer through sexual contact.*

Cancer is not a sexually transmitted disease [34].

5. Can the treatments be transmitted to my partner during sex?

*No, the treatments you are taking cannot be transmitted to your partner during sexual intercourse.*

6. Do I have to use contraception after the transplant?

*For women of childbearing age, effective contraception is recommended up to about 2 years after the transplant, and can be discussed on a case-by-case basis with your hematologist depending on the type of blood disease and the type of transplant. Indeed, some treatments may be incompatible with pregnancy.*

The teratogenic effects, whether confirmed or potential, of pre-transplant treatments, and certain therapies likely to be administered post-transplant are described [35,36]. Some drugs are not recommended or may have undetermined effects on the fetus such as methotrexate, some antifungals and cytotoxic agents.

Contraception may be advised by the gynecologist for a patient of childbearing age, in a manner similar to certain oncological situations. The minimum duration of 2 years post-transplant relies on the idea that this is the period with highest risk of relapse [31,32,33,34,35]. Contraception can be followed by the patient or his/her partner.

7. Am I at risk of being sterile?

*This subject is generally discussed before the transplant with your referring doctor who is obligated to answer your questions. A consultation with a reproductive specialist may have been offered to you to discuss your future fertility and to preserve your fertility, if necessary. Depending on the treatments you have received, the impact on your fertility may be different. You may be offered a check-up in due course. You can return to a reproductive specialist. In case of infertility or hypofertility and if there is a desire for a child, you may be offered appropriate treatment.*

For women of childbearing age, it is strongly recommended that a consultation with a doctor specialized in procreation be carried out and, when possible, an ovarian harvest may be considered in case of a highly gonadotoxic treatment [37,38]. The concept and the method of detection of residual disease on the ovarian sample should be discussed on a case-by-case basis depending on the hemopathy concerned [39]. Alternatives to ovarian harvesting are also to be discussed during this consultation [40,41].

It is essential to ask men about their desire to have children before starting chemotherapy, and to remember to systematically freeze their sperm if necessary. The question may be asked at the time of the pre-transplant consultation and some practitioners may realize that a sperm freezing was not performed at the time of the diagnosis of the hematological disease. A specialized opinion has to be taken to discuss the solution on a case-by-case basis, depending on the previous treatments received and the planned conditioning [42,43]. A remote post-transplant spermogram can be performed.

8. How do I know if I am menopausal?

*Depending on the treatments you have received, the impact on ovarian function remains variable. A consultation with a reproductive specialist will be proposed as well as a gynecological follow-up with the possibility of a treatment adapted to your situation.*

We recommend a gynecological consultation as part of a pre-transplant assessment, as well as regular post-transplant follow-up (see Annex S1 in Supplementary Materials) [44]. There may be a resumption of the menstrual cycle, with different frequencies, that is not necessarily correlated with fertility [45]. In this case, it may also be possible to consider preserving fertility, such as freezing isolated mature oocytes. Accordingly, consultation with a reproduction specialist may be important [33,35].

9. Are there any prohibited practices?

*There are no practices that are prohibited a priori. However, it is recommended to avoid potentially traumatic practices when the platelet count is too low. Some patients say that they often need to use lubricants, while others say that they need to boost their libido by playing erotic games with their partner.*

It is best to avoid potentially traumatic practices (sex toys, etc.), especially in case of thrombocytopenia less than 50 G/L or under anticoagulant treatment or long-term corticosteroid therapy (skin and/or mucous membrane fragility).

10. Are there any special hygiene measures?

*Intimate hygiene consists of a non-aggressive daily cleansing with a suitable mild soap, once a day. Other potential toilets should be carried out with clean water. It is recommended to wear cotton underwear and if possible, not to wear it at night. These recommendations also apply to your partner.*

11. I have problems with my erection and/or ejaculation, I can’t feel pleasure, I don’t feel like having sex. What can I do?

*These disorders are frequent and are independent of the feeling of love. They can have many reasons (treatments, hormones, fatigue, stress, anxiety, mood disorders, changes in body image, etc.), and can be transitory. If you feel you are having difficulties, it is important to find someone you know or a health professional with whom you feel confident to discuss the problem and find solutions, whether they be medication or other. Ask to meet with the multidisciplinary team who will refer you and your partner to the professional best able to support you.*

Fatigue is one of the most frequently mentioned symptoms on discharge from hospital without necessarily having a physical cause [46,47,48]. It can be the expression of a general, multifactorial illness that can impact sexual and emotional life. The supportive care team should be there to help the patient and restore confidence [49]. It is recommended to plan a consultation alone and/or with the partner. It is important to emphasize the need to communicate with the partner. A study carried out between couples showed a poor concordance between transplant survivors and partners in attitudes toward sexuality, satisfaction with sexual activity and causes of sexual dysfunction [15].

12. I have pain during intercourse.

*Desire, lubrication and pleasure can be affected by the treatments received. These complications may be the result of vaginal dryness or mucosal lesions, which are themselves linked to a side effect of the treatments, the occurrence of GVHD or another cause. A gynecological or urological consultation is recommended to provide you with therapeutic answers. It is essential to report any disorder, as well as other side effects of the allograft, in order to be referred to a consultation with a specialist. Solutions exist.*

It is essential to report any disorder, as well as the other side effects of the allogeneic transplant, in order to be referred to the right specialist. Desire, lubrication and pleasure can be impacted by the treatments received. These complications can be the result of vaginal dryness or mucosal damage. The incidence of genital GVHD is significant in both women and men. Genital GVHD can cause pain during sex [17,19,20]. It is necessary to prevent it (pre-transplant gynecologist consultation, lubricants, etc.) to detect and early treat genital GVHD (see Annex S1 in Supplementary Materials). In women, a narrowing (stenosis) or shortening of the vagina may be seen with cGVHD. The wall of the vagina may be less flexible: the use of dilators, the maintenance of sexual intercourse and the use of lubricants can prevent this process. In men, erectile dysfunction can be observed with or without cGVHD [21,23,50]. It may be the complete absence of an erection, including nocturnal or morning erection, or an insufficient erection in quality and/or duration, and consequently does not allow satisfactory intercourse. Changes can also be seen with ejaculation, which may be sometimes painful, diminished or absent. A consultation with a urologist and/or an andrologist may be necessary: a testosterone-type hormonal assessment may be requested as well as a standard recommended evaluation for any erectile dysfunction (cardiac assessment, LDL, HDL, triglycerides, PSA, blood sugar, diabetes balance, hypertension). It is advisable for men to have also urological and andrological follow-up [51]. Unlike women, this monitoring has not yet been even minimally implemented or standardized.

## 8. Conclusions

A multidisciplinary approach should be favored to treat the different aspects of the sexual and emotional life of allo-HCT patients. This approach must be organized and integrated into the general organization of patient care and therefore into the training of the healthcare team. In order to facilitate this management, it is necessary to systematize it, that is to say: to make it an integrated part of routine care, in the same way as the dosage of ciclosporin or the search for GVHD. For this, we offer this booklet for patients and this article for caregivers, as well as a proposal for gynecological care’s basic organization. This work was adapted from the recommendation of SFGM-TC workshops article [28]. There are still unresolved issues. In particular, unlike women, endocrinological follow-up of men is not at all systematic. A discussion should be established between endocrinologists, urologists, andrologists and sexologists to set up such a follow-up. Moreover, it is necessary for each center locally to constitute a network of correspondents for all the disciplines that were mentioned. However, due to the lack of social security coverage for certain paramedical consultations (sex therapists in particular), it may be difficult for patients to afford such a financial requirement, especially when follow-up is not done in the hospital setting. A reflection on the global management of the sexual and affective health of the patient after allogeneic transplantation should be undertaken taking into account the psychological, physical and financial aspects (creation of paramedical positions, support by the social security and healthcare assurance organizations for paramedical consultations, etc.).

## Figures and Tables

**Table 1:** Sexual dysfunctions after allogeneic HSCT.

Non-specific signs	
	Libido disorder
	Mood disorder
	Depression
	Asthenia
Signs related to premature ovarian failure in women	
	Irregular or absent periods
	Hot flashes
	Nocturnal sweat attacks
	Vaginal dryness
	Osteoporosis
	Cardiovascular risk
	Hair loss
	Infertility
	Dyspareunia, vaginitis
	Urethritis, cystitis
Signs related to genital GVHD in men	
	Adhesions in the blood vessels of the penis
	Skin sensitivity
	Arterial insufficiency with dysfunction of libido, erection and ejaculation
Signs related to genital GVHD in women	
	Dysuria
	Dyspareunia
	Inability to have sexual intercourse
	Vulvar dryness
	Vulvar redness
	Pain on touching the labia
	Ulcerations
	Inflammation of the genital mucosa
	Reduction of vaginal elasticity
	Leukokeratosis
	Vaginal adhesions
	Vaginal stenosis

## Data Availability

Not applicable.

## Supplementary Materials

The following supporting information can be downloaded at: https://www.mdpi.com/article/10.3390/jcm11051196/s1, Annex S1: Recommended Follow-up Schedule following allo-HCT; Annex S2: The Patient Booklet.
