# Supplementary material for: Sexual and Emotional Health after Allogeneic Hematopoietic Cell Transplantation: A Comprehensive Review and Guidelines from the Francophone Society of Bone Marrow Transplantation and Cellular Therapy (SFGM-TC)
Source: J Clin Med. 2022 Feb 23;11(5):1196. doi: 10.3390/jcm11051196 (PMC8911485; doi:10.3390/jcm11051196)
Supplement: Supplementary file 1 [file jcm-11-01196-s001.zip › jcm-1555911-supplementary.pdf]

Annex S1.

FREQUENCY OF CONSULTATIONS (cs)

First cs at Day+100, then every 3 months for 2 years, then three times a year for 2-3 years, then biannual.
The frequency of cs should be higher if HPV, GVHD or intolerance to HRT.

FREQUENCY OF EXAMS

1- Mammogram at baseline then every 18 months,
or bilateral breast ultrasound if young woman, Fanconi's disease or other haematological pathologies contraindicating radiation.

2- Cervical screening at two months, then once a year
Except if Papillomavirus:
- External genital warts: destruction by liquid nitrogen or imiquimod (ALDARA®) = immunomodulator, duration not limited
- Smear abnormalities: ASCUS or ASGUS, suspected dysplasia, mild, moderate or severe
> How to proceed: Disinfection by ovules (type POLYGYNAX®)
Colposcopy after 3 to 6 months, depending on the degree of dysplasia
Cervical biopsy, according to pathology results.

3- Osteodensitometry: at baseline (reference), then depending on the results and eventual treatment introduced.

4- Pelvic ultrasound: (reference)
Normal or atrophic uterus
Normal or atrophic ovaries (follicles)
Monitoring of patients on hormone replacement therapy (HRT)

PROCEDURE FOR CONSULTATIONS

1- First gynecological examination

1-1. Questioning

Gynecological and obstetrical history
First menstrual period
Number of pregnancies (VTP, spontaneous abortion, living children), modalities of delivery or breastfeeding
Hormonal contraception, IUD, condoms, local etc.
Date of menopause, general or local HRT before transplantation
Vulvar functional signs: dyspareunia, burning, itching
Sexual intercourse before and since transplant, with or without condom
General history
Medical: haematological disease, thromboembolic events, asthma, allergy etc.
Surgical
Other GVHD impairments: eye, skin, scalp, mouth, digestive, pulmonary

1-2. Examination

General

Skin, adenopathies
Gynecological
Breast and axillary lymph nodes palpation
Vulva: normal, dryness, redness, pallor (elasticity), lack of consistency
Labia minora: present, atrophic, missing
Clitoris: normal, partially or completely hooded
Vestibule: pale, red, permeable, narrow or stenosed (vestibular synechia)
Vagina: permeable, partial or total stenosis
Cervix: accessible to smear or not
Search for warts in all genital areas (vulva, vaginal, anal, oral)

1-3. Procedure

Reference mammogram and/or breast ultrasound, MRI if required
Baseline bone densitometry
Pelvic ultrasound
Normal or atrophic uterus
Normal or atrophic ovaries (follicles)
Only local estrogen treatment at first: vaginal ovules every day for 15 days then thrice a week, and cream: four tubes per month for two months.
This treatment can be introduced immediately after the aplastic period.

2- Second gynecological consultation

2-1. Hormone replacement therapy (HRT)

No contraindication to HRT (normal mammogram, normal smears, normal pelvic ultrasound, no thromboembolic event (phlebitis, pulmonary embolism)
Introduction of percutaneous estradiol gel (ESTREVA® gel), in gradually increasing doses: one dose for 10 days, then two doses for 10 days to reach three doses as long-term treatment, depending on patient's tolerance (mastodynia, pelvic pain, nausea...).
Macroprogestin, e.g. normegestrol acetate, can be combined, taking into account the current prescription restrictions due to the risk of meningioma.
As soon as possible, reassessment of HRT to a lesser dose of progestin or other treatment appropriate to the patient or clinical situation, depending on the need for contraception.

2-2. Human Papilloma Virus (HPV)

From the first gynecological consultations: offer the HPV vaccine, regardless of vaccination status and age: GARDASIL 9 (and not GARDASIL)
before 14 y.o.: 2 doses 6 to 13 months apart
from 14 y.o.: 3 doses according to a M0, M2 and M6 schedule
GARDASIL 9 vaccination is also recommended for boys and young men.
In case of HPV infection: perform HPV typing and especially request a Pap smear cytology after prolonged disinfection with ovules, colposcopy if necessary.
Oncogene HPV request very attentive cytological follow-up at each gynecological consultation.
Warn about the risks of sexually transmitted diseases in case of unprotected sex and think of doing a systematic screening or in case of symptoms
(HIV, Chlamydiae trachomatis, Neisseiria gonorrheae, Trichomonas vaginalis, hepatitis)

2-3. GVHD

Biopsy is unnecessary because histological confirmation is late and the diagnosis of gynecologic GVH is clinical.
It is more harmful not to treat gynecological GVHD than to overtreat.
As soon as there is a strong suspicion of gynecological GVH, it is necessary to see the patients closely, especially in the early forms which can evolve very quickly.
Treatment with corticosteroids of Dermoval® type (class 4 steroids) and intra vaginal corticosteroid foam
Evaluation after treatment:
Unchanged

Improvement
Restitution ad integrum
Surgery immediately or after estrogen treatment

Annex S2: The Patient Booklet

Sexual and emotional life after transplantation: Let's talk about it?

Since January 2002, the WHO has declared that "Sexual health is an integral part of overall health, well-being and quality of life (...)".

http://www.who.int/reproductivehealth/topics/gender_rights/sexual_health/en/

All the events surrounding the hematopoietic stem cell transplantation represent a stress that may have an impact on the resumption of sexual life in the period of return home. Some changes may be temporary, others more lasting, both in men and women.

There are many factors involved:

- Psychological: stress, anxiety, depression, loss of self-esteem, changes in self-image, fears, hyper-vigilance, inability to let go, changes in relationships as a result of the disease
- Physiological: weight loss or gain, hair loss, asthenia, vaginal dryness, sterility, dryness of the mucous membranes in men, hormonal dysfunctions, a feeling of great fatigue, lack of energy,

The intimate relationship remains something alive to be reinvested with body, heart and mind and throughout life sexuality varies in intensity, desire and erotic scenarios.

Whether you are single or in a relationship, the aim of this document is to provide you with a wide range of answers, which obviously cannot be exhaustive, as everyone's experience is unique. This information is presented in the form of answers to certain preconceived ideas or frequently asked questions.

We hope to encourage discussion with your partner and/or health care team.

1- Can I resume intercourse? When can I start having sex again?

Sexual intercourse can be resumed as soon as you return home. It is important to emphasize that sex is not limited to penetration. It is also about sensual caresses, tenderness, touching, communication, not forgetting masturbation and kissing. Patients often mention a period of gradual resumption of sexual life after the disease.

2- I am afraid to resume sexual relations, is this normal?

After a long period of hospitalization and isolation, associated with very strict hygiene measures, returning home can be a source of fear, questioning and anxiety which can impact your intimate life. An adaptation phase is often necessary after a cessation of sexual activity, while going through such an intense process as a transplant. Communication with your partner is essential. Ask your medical team questions if your fear persists and becomes a major concern for you. No one can imagine what is going on in your head and in your partner's head. Just talk about it. There may be new forms of erotic and sexual relationships that may be temporary, things are never set in stone.

3- Are there any particular infectious risks associated with resuming sexual activity? "Do I need to use a condom?

The risks of sexually transmitted infections (STIs) are the same as in the general population. Thus, it is recommended to use a condom in case of multiple partners or unknown HIV status, as well as in case of a history of genital warts in any partner. It is also recommended that condoms be used for oral or anal sex for as long as immunosuppression lasts.

In the event of genital or oral lesions, it is necessary to talk to the attending or referring physician who will be able to guide you.

4- Is there a risk of contagion of the cancer to my partner by sexual means?

No, there is no risk of contagion of cancer through sexual contact.

5- Can the treatments be transmitted to my partner during sex?

No, the treatments you are taking cannot be transmitted to your partner during sexual intercourse.

6- Do I have to use contraception after the transplant?

For women of childbearing age, effective contraception is recommended up to about 2 years after the transplant, and can be discussed on a case-by-case basis with your hematologist depending on the type of blood disease and the type of transplant. Indeed, some treatments may be incompatible with pregnancy.

7- Am I at risk of being sterile?

This subject is generally discussed before the transplant with your referring doctor who is obligated to answer your questions. A consultation with a reproductive specialist may have been offered to you to discuss your future fertility and to preserve your fertility, if necessary. Depending on the treatments you have received, the impact on your fertility may be different. You may be offered a check-up in due course. You can return to a reproductive specialist. In case of infertility or hypofertility and if there is a desire for a child, you may be offered appropriate treatment.

8- How do I know if I am menopausal?

Depending on the treatments you have received, the impact on ovarian function remains variable. A consultation with a reproductive specialist will be proposed as well as a gynecological follow-up with the possibility of a treatment adapted to your situation.

9- Are there any prohibited practices?

There are no practices that are prohibited a priori. However, it is recommended to avoid potentially traumatic practices when the platelet count is too low.

Patients say that they often need to use lubricants, others say that they need to boost their libido by playing erotic games with their partner.

10- Are there any special hygiene measures?

Intimate hygiene consists of a non-aggressive daily cleansing with a suitable mild soap, once a day. Other potential toilets should be carried out with clean water. It is recommended to wear cotton underwear and if possible, not to wear it at night. These recommendations also apply to your partner.

11- I have problems with my erection and/or ejaculation, I can't feel pleasure, I don't feel like having sex, what can I do?

These disorders are frequent and are independent of the feeling of love. They can have many reasons (treatments, hormones, fatigue, stress, anxiety, mood disorders, changes in body image, etc.), and can be transitory. If you feel you are having difficulties, it is important to find someone

you know or a health professional with whom you feel confident to discuss the problem and find solutions, whether they be medication or other. Ask to meet with the multidisciplinary team who will refer you and your partner to the professional best able to support you.

12- I have pain during intercourse

Desire, lubrication and pleasure can be affected by the treatments received. These complications may be the result of vaginal dryness or mucosal lesions, which are themselves linked to a side effect of the treatments, the occurrence of GVHD or another cause. A gynecological or urological consultation is recommended to provide you with therapeutic answers. It is essential to report any disorder, as well as other side effects of the allograft, in order to be referred to a consultation with a specialist. Solutions exist.
